# *NPR1* as a transgenic crop protection strategy in horticultural species

**DOI:** 10.1038/s41438-018-0026-1

**Published:** 2018-03-20

**Authors:** Katchen Julliany P. Silva, Nasser Mahna, Zhonglin Mou, Kevin M. Folta

**Affiliations:** 1 Horticultural Sciences Department, University of Florida, Gainesville, FL 32611 USA; 2 Department of Horticultural Sciences, University of Tabriz, Tabriz, Iran; 3 Department of Microbiology and Cell Science, University of Florida, Gainesville, FL 32611 USA; 4 Graduate Program in Plant Molecular and Cellular Biology, University of Florida, Gainesville, FL 32611 USA

## Abstract

The *NPR1* (*NONEXPRESSOR OF PATHOGENESIS RELATED GENES1*) gene has a central role in the long-lasting, broad-spectrum defense response known as systemic acquired resistance (SAR). When overexpressed in a transgenic context in *Arabidopsis thaliana*, this gene enhances resistance to a number of biotic and abiotic stresses. Its position as a key regulator of defense across diverse plant species makes *NPR1* a strong candidate gene for genetic engineering disease and stress tolerance into other crops. High-value horticultural crops face many new challenges from pests and pathogens, and their emergence exceeds the pace of traditional breeding, making the application of *NPR1*-based strategies potentially useful in fruit and vegetable crops. However, plants overexpressing *NPR1* occasionally present detrimental morphological traits that make its application less attractive. The practical utility of NPR-based approaches will be a balance of resistance gains versus other losses. In this review, we summarize the progress on the understanding of NPR1-centered applications in horticultural and other crop plants. We also discuss the effect of the ectopic expression of the *A*. *thaliana NPR1* gene and its orthologs in crop plants and outline the future challenges of using *NPR1* in agricultural applications.

## Plant immunity: Engineering for enhanced resistance

Introducing the plant immunity gene *NPR1* into fruits and vegetables may help to protect them against diseases and pests, which are emerging and migrating faster than resistance can be developed using traditional breeding methods. Pandemics currently threaten crops such as banana, cacao, citrus, and avocado, and genetic engineering may offer a rapid, low-input alternative for crop protection. Kevin Folta at the University of Florida and co-workers reviewed the use of *NPR1* to boost immunity in horticultural plants. *NPR1* was first tested in high-acreage crops such as rice, and it is now being tested in strawberry, grapevine, tomato, carrot, cotton, citrus, tobacco, and many others. Introducing *NPR1* usually improves resistance, and researchers are testing ways to minimize undesirable side effects, such as reduced yield. Transgenic approaches show promise for helping plants adapt to modern stresses.

## Introduction

Plant pathogens challenge profitable production, and in some cases threaten entire industries. Current plant pandemics in banana, citrus, avocado, and cacao mark just a few dire examples where rapid and durable solutions are desperately needed. These visible cases represent just the edge of a much broader problem, as plant pathogens are shuttled worldwide with borderless travel and on winds of weather extremes. At the same time, our arsenal of chemical approaches to quell disease presentation is antiquated and slow to evolve. There also is the desire to limit production inputs, generating savings for farmers and benefits to the environment.

Genetic engineering solutions have proven successful in mitigating the effects of plant disease in the laboratory and a limited number of field trials, in a number of plant species. A series of reports have described the effect of *NPR1* (*NONEXPRESSOR OF PATHOGENESIS-RELATED GENES1*) overexpression on disease progression and symptom presentation. *NPR1* is a central regulator of plant defense response. It stands out as a conspicuous target for use in a transgenic context, as a plant gene inappropriately expressed to induce an enhanced response is an intuitive target for engineering disease-tolerant plants.

Overexpression of the *Arabidopsis thaliana NPR1* gene (*AtNPR1*) or its orthologs has been shown to enhance resistance to biotrophic and necrotrophic fungal, viral, and bacterial pathogens in a number of horticultural crop plants, including grape, carrot, tomato, apple, citrus, tobacco, and strawberry. These specialty crop applications occurred after applications in high-acreage agronomic crops like rice, wheat, soybean, peanut, and potato^1–15^. These trials suggest that similar defense mechanisms exist across a range of plant species, making *AtNPR1* and its orthologs desirable candidate genes for transgenic manipulation in crops for enhanced disease resistance^3,16–18^.

In this review, we provide an overview of reports that have altered disease and pest tolerance by manipulating *NPR1* expression levels, describing the work translated to agronomic crops, and then the reports arising from horticultural crop species.

## Plant defense mechanisms

Plants cope with pathogen infection with sophisticated chemical, biochemical, and mechanical barrier systems. These systems combined are the basis of plant innate immunity, and allow plants to survive, grow properly, and produce appreciable crop yields. In contrast to mammals, plants did not evolve mobile defender cells and somatic adaptive immune system. Instead plants rely on the innate immunity of each cell, as well as on systemic signals emanating from infection site to mobilize a defense response^19,20^. Fortunately, pathogen recognition and defense activation occur rapidly. Potentially infecting pathogens must first overcome several physical barriers on the plant surface, such as wax layers, rigid cell walls, cuticular lipids, trichomes, hairs or spines, leaf veins, odd stomatal aperture shapes, and antimicrobial enzymes or secondary metabolites^21,22^. These morphological structures and chemical barriers represent the first line of plant defense. These features play an important primary role in disease resistance by inhibiting or delaying the advance of pathogens into invasive states^23–25^. A successful infection requires breaking initial barriers and efficiently countering a cascade of plant defense responses generated by a suite of mobilized biochemical activities^26^.

Pathogen signatures are also recognized by plants at a molecular level. The plant immune system can be summarized in three steps, as described by Jones and Dangl^27^ as well as Zipfel^28^. First, pathogen- or microbial-associated molecular patterns (P/MAMPs) are recognized by pattern recognition receptors, resulting in pathogen- or microbial-triggered immunity (P/MTI). Second, virulent effectors are deployed by successful pathogens to interfere with P/MTI, which results in effector-triggered susceptibility. After a given effector is specifically recognized by one of a class of proteins called the nucleotide binding site-leucine-rich repeat proteins, which activate effector-triggered immunity, resulting in disease resistance. This second layer of immunity often culminates with a hypersensitive response (HR), comprised of programmed cell death^29^ at the infection site to isolate the pathogen, followed by the upregulation of defense responses such as accumulation of pathogenesis-related (PR) proteins in distal tissues. This response, known as systemic acquired resistance (SAR), protects plants from secondary infection by activating multiple signaling pathways and inducing systematic responses to confront pathogen infection^30,31^.

## SAR and *PR* genes

SAR is a well-known plant resistance mechanism induced upon pathogen infection. It is activated by the accumulation of the signaling molecule salicylic acid (SA, 2-hydroxybenzoic acid) and the coordinated induction of *PR* genes that encode proteins meeting pathogen attack^32,33^. Systemic accumulation of SA at the onset of SAR has been well characterized in the model plant *A*. *thaliana*. The key regulator of SAR is the NPR1 protein. It contains BTB/POZ and ankyrin repeat domains, which mediate NPR1 interaction with other proteins and help the protein to associate with the promoters of *PR* genes, activating their expression^34,35^. In the absence of infection, NPR1 is predominantly oligomeric and partitioned to the cytoplasm. During pathogen infection or SA treatment, the NPR1 complex disarticulates, and monomeric proteins are transported into the nucleus. In the nucleus, NPR1 interacts with the TGA family of basic leucine zipper transcription factors^35^, which in turn upregulate the transcription of *PR* genes, and help confer resistance to secondary infection^34,36–38^.

## Use of *AtNPR1* and its orthologs for crop improvement

### Heterologous expression of AtNPR1 in agronomic crops

The *AtNPR1* protein triggers the activation of defense genes upon infection, so many groups sought to test the hypothesis that promiscuous expression of the *AtNPR1* gene would result in enhanced resistance to disease in crop plants. The hypothesis was generally tested by placing the *AtNPR1* cDNA downstream of the CaMV 35S promoter. This approach has resulted in some positive effects on disease resistance (Table 1). While the focus of this review is horticultural crops, it is necessary to start with the application of *AtNPR1* in agronomic crops because that is where the first translational experiment was performed.

**Table 1:** Transgenic crop plants ectopically expressing *AtNPR1*

Plant species	Resistance/tolerance (caused by)	Abnormal phenotype	Reference
Canola	Bacterial disease (*P.syringae*)	a	Potlakayala et al.^44^
Carrot	Sclerotinia rot (*S. sclerotiorum*) Gray mold (*B. cinerea*) Black rot (*A.radicina*) Bacterial leaf blight (*X*. *hortorum* pv. *carotae*) Powdery mildew (*Erysiphe heraclei*)	a	Wally et al.^7^
Citrus	Citrus canker (*X*. *citri* ssp. *citri*.) Citrus greening (Huanglongbing)	a	Zhang et al.^9^ Dutt et al.^50^ Boscariol-Camargo et al.^48^
Cotton	*Verticillium* wilt (*V*. *dahliae*) *Fusarium* wilt (*F*. *oxysporum* f. sp. *vasinfectum*) Seedling damping-off (*R*. *solani*) *Alternaria* leaf spot (*A*. *alternata*) Nematode (*R*. *reniformis*) Black root rot (*T*. *basicola*)	a	Kumar et al.^45^ Parkhi et al.^46^^,^^47^
Rice	Bacterial blight (*X*. *oryzae pv*. *oryzae*) Blast disease (*M*. *oryzae*) Bakanae disease (*F*. *verticillioides*) Bacterial foot rot (*E*. *chrysanthemi*) Sheath blight (*R*. *solani*)^a^	Lesion-mimic cell death Accumulation of H_2_O_2_ around lesions Susceptibility to the rice yellow mottle virus Growth retardation, reduced height and smaller seeds, with consequent lower productivity; and development of spontaneous lesions in controlled conditions	Chern et al.^17^^a^ Fitzgerald et al.^39^ Quilis et al.^40^ Molla et al.^42^^a^
Plant species	Resistance/tolerance (caused by)	Abnormal Phenotype	Reference
Soybean	Root-knot nematodes (Meloidogyne spp.) Cyst nematode (*H*. *glycines*)	a	Youssef et al.^13^ Matthews et al.^12^
Strawberry	Anthracnose (*C*. *acutatum*) Crown rot (*C*. *gloeosporioides*) Powdery mildew (*P*. *aphanis*) Angular leaf spot (*X*. *fragariae*)	Shorter plants, reduced canopy size and density No production of runners and fruits	Silva et al.^11^
Tobacco	Common cutworm larvae (*S*. *litura*) Root-rot nematode (*M*. *incognita)* polyethylene glycol and oxidative stress tolerance	a	Meur et al.^6^ Priya et al.^52^ Srinivasan et al.^53^
Tomato	Bacterial wilt (*R*. *solanacearum*) Gray leaf spot (*S*.*solani*). Bacterial spot (*X*. *campestris* pv. *vesicatoria*)	Susceptibility to *Fusarium* wilt (*F*. oxysporum f. sp. *lycopersici*)	Lin et al.^3,39^
Wheat	*Fusarium* head blight (*F*. *graminearum*)	Susceptibility to *Fusarium* seedling blight (*Fusarium* spp.)	Makandar et al.^4^^a^
Wheat	*Fusarium* head blight (*F*. *graminearum*)	Susceptibility to *Fusarium* seedling blight (*Fusarium* spp.)	Gao et al.^43^

^a^No abnormal/no reported phenotype

The first case of *AtNPR1* overexpression in an agronomic crop was reported by Chern et al.^17^, who observed that promiscuous expression of *AtNPR1* in rice led to enhanced resistance to bacterial blight, caused by *Xanthomonas oryzae* pv. *oryzae*. Disease resistance segregated among individual T1 plants, which approximated *AtNPR1* steady-state transcript levels. As in Arabidopsis, no obvious morphological changes were observed^16^. Agronomic traits of rice *AtNPR1*-transgenic lines were not affected and most plants were fertile.

However, a subsequent report in rice confirmed that while *AtNPR1* overexpression enhanced resistance to *X*. *oryzae* pv. *oryzae*, it caused detrimental side effects to plant growth^39^. Transgenic rice exhibited lesion-mimic cell death, hydrogen peroxide (H_2_O_2_) accumulated significantly around lesions, and plants accumulated lower levels of free SA. These phenotypes were heritable and positively correlated with steady-state transcript levels of the *AtNPR1* transgene and endogenous rice defense genes. Quilis et al.^40^ also reported that *AtNPR1* has both positive and negative regulatory roles in mediating defense responses in rice against biotic and abiotic stresses. The *AtNPR1* transgene conferred resistance to the fungal pathogens *Magnaporthe oryzae* and *Fusarium verticillioides*, which cause blast and bakanae diseases, respectively, and to the bacterial pathogen *Erwinia chrysanthemi* that causes foot rot disease. The transgenic plants, however, showed susceptibility to rice yellow mottle virus. Ectopic *AtNPR1* expression also resulted in salt and drought stress sensitivity. Unlike Arabidopsis and tobacco^41^, rice plants accumulated very high levels of endogenous SA, and these levels did not change after pathogen infection. *AtNPR1* overexpression also affected rice yields under controlled conditions. In the greenhouse, transgenic rice plants overexpressing *AtNPR1* showed slower growth, reduced height, and smaller seeds. In the growth chamber, *AtNPR1* plants developed spontaneous lesions. Another report in rice showed that green-tissue-specific expression of *AtNPR1* in rice resulted in increased resistance to sheath blight disease (caused by *Rhizoctonia solani*) with no phenotypic abnormalities. This strategy was considered an improvement overexpression from a constitutive promoter, where some deleterious effects were observed^42^.

*AtNPR1* was also ectopically expressed in wheat in order to assess effects on disease resistance^4^. Transgenic plants exhibited increased resistance to head blight (FHB) caused by *F*. *graminearum* Schwabe (teleomorph *Gibberella zeae* [Schwein.] Petch) and demonstrated stable inheritance of resistance for four generations. In another study, accumulation of the *AtNPR1* transcript positively affected wheat yield^39^. *PR1* transcripts accumulated faster and to higher levels in the transgenic plants. Increased wheat resistance to FHB and *Fusarium* seedling blight (*Fusarium* spp.) was also later reported by Gao et al.^43^.

*AtNPR1-*expressing canola (*Brassica napus* cv. Westar) plants displayed resistance to *Pseudomonas syringae*^44^. The transgenic plants exhibited no abnormal phenotypes, and the *AtNPR1* transcript levels did not correlate with disease resistance.

Constitutive expression also led to effects against other stressors, such as nematodes. The search for increased resistance to nematodes in soybean spurred the examination of several Arabidopsis genes related to SA and JA synthesis and signaling^13^. Transgenic *AtNPR1*-soybean plants were resistant to root-knot nematodes (RKN) (*Meloidogyne* spp.). The number of RKN galls per plant was reduced in transgenic plants. In another study, Matthews et al.^12^ generated plants with resistance to soybean cyst nematode (SCN) (*Heterodera glycines*). Promiscuous *AtNPR1* overexpression resulted in the lowest SCN cyst index, suggesting that this gene is a strong candidate for plant genetic engineering to confer nematode resistance.

### Heterologous expression of *AtNPR1* in horticultural crops

The *AtNPR1* gene also triggered resistance to several tropical diseases in tomato^3^. Transgenic tomato plants with the highest *AtNPR1* transcript accumulation exhibited enhanced resistance to bacterial wilt (caused by *Ralstonia solanacearum*), *Fusarium* wilt (caused by *F*. *oxysporum*. sp. *lycopersici*), gray leaf (caused by *Stemphylium solani*), and bacterial spot (caused by *X*. *campestris* pv. *vesicatoria*). Plants exhibited normal morphologies and horticultural traits for at least four generations. Lines with medium or low levels of *AtNPR1* expression showed similar or slightly lower resistance to *Fusarium* wilt when compared to wild-type plants.

*AtNPR1-*expressing carrot plants were resistant to multiple pathogens^7^. Transgenic lines were phenotypically normal compared to non-transformed controls. Three necrotrophic foliar pathogens, *Sclerotinia sclerotiorum*,* Botrytis cinerea*, and *Alternaria radicina*, a root pathogen, *A*. *radicina*, a foliar infecting bacteria, *X*. *hortorum* pv. *carotae*, and a biotrophic fungus, *Erysiphe heraclei*, were tested. Disease symptoms were reduced in all treatments. The best results were observed for *X*. *hortorum* and *E*. *heraclei*, which had disease reduced by 80% and 90%, respectively. Transcripts corresponding to carrot *PR* genes were not elevated in *AtNPR1*-overexpressing carrot plants, suggesting enhanced resistance did not correlate with constitutive induction of SAR.

Other examples of broad-spectrum resistance have been described in cotton^45–47^. *AtNPR1*-expressing cotton lines were resistant to the fungi *V*. *dahliae*^46^, *F*. *oxysporum* f. sp. *vasinfectum, R*. *solani*, and *A*. *alternata* and to the nematode, *Rotylenchulus reniformis*^47^. The transgenic plants displayed normal growth and development but showed typical discoloration symptoms. *AtNPR1*-expressing cotton also showed resistance to *Thielaviopsis basicola*, the causal agent of black root rot^45^. The transgenic plants displayed significantly higher shoot and root weights, longer shoots, and eventually improved yields. They also exhibited faster and stronger induction of many *PR* genes in roots, including *PR1* and *LIPOXYGENASE1*.

Perennial fruit tree crops, such as citrus, have also been transformed with *AtNPR1* and their growth and development are largely unaffected, while conferring tolerance to disease. Zhang et al.^9^ showed that *AtNPR1* in “Duncan” grapefruit and “Hamlin” sweet orange conferred increased resistance to citrus canker (caused by *X*. *citri* ssp. *citri*.). Basal levels of SA were not altered in any of the tested lines, and the SAR marker gene in citrus, *PR2*, was not constitutively expressed, suggesting that *AtNPR1* ectopic expression did not induce a constitutive defense response. A similar effect was shown in *AtNPR1*-overexpressing sweet orange lines by Boscariol-Camargo et al.^48^. The most conspicuous effect was an increase in the accumulation of *enhanced disease susceptibility1* (*EDS1*), *PR1* and *PR2* transcripts 12–24 h after inoculation by *Xcc*. This induction was more noticeable for *PR1*, which showed a 21,000-fold increase (likely due to low transcript levels in the non-induced plant) in transgenic and non-transgenic lines in relation to the non-inoculated control plants. The authors proposed that *AtNPR1* induces resistance to *Xcc* through a priming mechanism, essentially increasing *EDS1* and *PR* transcript accumulation to a higher level after pathogen inoculation.

Ectopic expression of *AtNPR1* under the control of the bi-directional dual promoter complex with enhanced promoter activity^49^ resulted in sweet orange lines with tolerance to Huanglongbing (also called citrus greening; caused by *Liberibacter asiaticus*). An equivalent outcome was witnessed when a phloem specific *A*. *thaliana SUC2* (*AtSUC2*) promoter was implemented. The transgenic plants had fewer disease symptoms and a few lines remained symptom-free after 36 months in a field with high disease pressure and highly symptomatic controls^50^.

The diploid strawberry (*F*. *vesca* L. Hawaii 4’) has also been engineered with a *35S::AtNPR1* cassette. Transgenic plants showed increased resistance to anthracnose (caused by *C*. *acutatum*), crown rot (caused by *C*. *gloeosporioides*), powdery mildew (caused by *P*. *aphanis*), and angular leaf spot (caused by *X*. *fragariae*). The disadvantage was an increased instance of undesirable traits observed during plant growth and development. The transgenic plants were shorter than non-transformed controls, and canopy size and density were reduced. In addition, all lines formed flowers but most did not produce runners, and fruit production was low^11^. These findings suggest that this strategy, while holding promise against disease, would benefit from a more nuanced expression approach, perhaps overexpressing the transcript in leaves or fruits only.

Constitutive expression of *AtNPR1* transcripts has also been investigated for effects on plant response to insect feeding^6^. Transgenic tobacco plants expressing *AtNPR1* were challenged with the herbivore *Spodoptera litura*, the common cutworm larvae. *AtNPR1*-tobacco plants were generally more resistant to herbivore feeding and early larval population growth. The increased resistance to *S*. *litura* was further investigated and a correlation with increased induction of serine protease inhibitors was observed. These compounds can slow down the digestion of ingested plant tissues in the insect gut, severely affecting larval growth^51^. *AtNPR1*-tobacco plants were also more resistant to the root-knot nematode (*M*. *incognita*)^52^. High *AtNPR1* transcript levels correlated with a 50–60% reduction in root galls and eggs-masses in infected roots and resulted in constitutive expression of *PR* genes. Another study reported that *AtNPR1*-tobacco displayed enhanced oxidative stress tolerance and did not suffer from polyethylene glycol stress^53^.

Heterologous expression of *AtNPR1* in various horticultural crops shows the potential to generate transgenic lines with increased broad-spectrum disease and pest resistance in most cases. Although poor public acceptance of genetically engineered plant products remains a challenge, the public may view cisgenic/intragenic approaches more favorably. While the Arabidopsis gene has shown promise in these trials, the native NPR1 may have a more intimate association with the inductive resistance mechanisms and provide better responses without the collateral effects.

### *AtNPR1* orthologs in agronomic crops

NPR1 appears to be functionally conserved across an array of agronomically relevant crops, spanning a diversity from rice to coconut palm^54^. Orthologs of *AtNPR1* have been cloned and characterized in many crop plants. While tremendous potential exists in horticultural crops, the first breakthrough and best characterization have been on large-scale commodity crops like rice, which is where early translational work began^1^. The closest match, *Os*NPR1/NH1, shares 46% identity and 60% similarity with the *At*NPR1 protein. The true orthology was confirmed when complementing the Arabidopsis *npr1* mutant^8^. *NH1* overexpression conferred resistance to the bacterial pathogen *X*. *oryzae pv*. *oryzae* and constitutively high accumulation of *PR* transcripts^1^, similar to what was observed upon *AtNPR1* overexpression in rice^17^. This finding suggests that NPR1-mediated disease resistance signaling pathways are similar in rice and Arabidopsis. Some negative phenotypes were reported. *NH1* plants displayed smaller stature, had lower fresh weight, and narrower or smaller leaves^1^. Under controlled conditions, transgenic plants had delayed growth and old leaves tended to senesce faster compared to wild-type plants. They also failed to develop additional tillers, spontaneously developed lesion-mimic spots, and showed more obvious HR-like responses after being challenged with *X*. *oryzae* pv. *oryzae*. NH1 may mediate antagonistic crosstalk between SA- and JA-dependent pathways in rice. Enhanced herbivore susceptibility in transgenic plants was also observed. Together with *NH1*, two additional *AtNPR1* homologous genes were found in rice (*OsNPR2/NH2* and *OsNPR3/NH3*). They were found to be induced by rice bacterial leaf blight (caused by *X*. *oryzae* pv. *oryzae*), rice blast (caused by *Magnaporthe grisea*), and the defense-related compounds benzothiadiazole (BTH; a synthetic inducer of SAR), methyl jasmonate (MeJA), and ethylene (ET). Chern et al.^55^ illustrated through co-expression analyses that *NH1* and *NH3* may have a common role in rice immunity. However, they have different co-expression patterns with *negative regulator of resistance*(*NRR*)* homolog1* (*RH1*) and *RH2* genes, as *NH1* co-expresses with *RH1* and *RH2* while *NH3* exhibits contrasting expression patterns.

A role for *NH1* against rice blast disease was demonstrated using a genome-wide analysis of BTH-responsive genes^56^, BTH-inducible blast resistance was compromised in plants where *NH1* transcripts were suppressed, and enhanced in *NH1*-overexpressing rice plants. This study also showed that photosynthetic activities are reduced by SA signaling. The transcripts corresponding to photosynthesis-related genes and chloroplastic ribosomal genes decrease in abundance when the SA signaling pathway is activated. *NH1*-overexpressing rice plants also showed enhanced resistance to *M*. *oryzae* and increased steady-state transcript levels of defense genes including *PR-1a*^57^.

The mustard (*Brassica juncea*) *NPR1*, *BjNPR1*, was cloned for assessing defense induction patterns upon chemical treatment and powdery mildew infection^58^. The amino acid sequence of *Bj*NPR1 shows 98% identity to *B*. *napus* NPR1 (*Bn*NPR1) and 69% identity to AtNPR1. The protein bears all the important functional domains, including the ankyrin repeats and the BTB-POZ domains. *BjNPR1* was constitutively expressed at low levels but was strongly induced by exogenous application of SA and upon *E*. *cruciferarum* infection. Moreover, complementation tests of *BnNPR1* in Arabidopsis *npr1* mutants showed restored SA-dependent induction of *PR-1* genes and enhanced basal defense, as well as provided systemic acquired resistance against *P. syringae*^44^. Additionally, *AtNPR1* and *BnNPR1* overexpression in transgenic *B*. *napus* effectively enhanced basal resistance against *P. syringae* with no obvious developmental abnormalities^44^, verifying the functional conservation of NPR1 between *A*. *thaliana* and *B*. *napus*.

Liu et al.^59^ used a virus-induced gene silencing (VIGS) system to test the role of the tobacco *required for MLA12 resistance1, EDS1*, and *NPR1* homologous genes in N-mediated resistance to tobacco rattle virus (TRV). Silencing of these genes compromised N function, suggesting that these genes play essential roles in the N-mediated resistance pathway. Further, characterization of cotton *NPR1* (*GhNPR1*) suggested that this *AtNPR1* ortholog is critical for activation of plant defense responses^60^. The predicted amino acid sequence exhibited 52.98%, 52.32% and 54.98% similarity to NPR1 from *A*. *thaliana, B*. *juncea* and *N*. *tabacum*, respectively. The GhNPR1 protein also contains an ankyrin repeat domain and a BTB/POZ domain, which are highly conserved among all NPR1 proteins and involved in protein–protein interactions^60^. Transcripts of *GhNPR1* could be markedly induced by SA, MeJA, and ET treatment, and by inoculation of *F*. *oxysporum* f. sp. *vasinfectum* and *X*. *campestris* pv. *malvacearum*, suggesting that *GhNPR1* is involved in response to biotic and abiotic stresses and may be critical for activation of defense responses in cotton. The soybean *GmNPR1-1* and *GmNPR1-2* genes are orthologous to *AtNPR1* and can enhance broad-spectrum resistance in soybean^61^. Both *GmNPR1* genes complemented the Arabidopsis *npr1-1* mutant and the transgenic plants were able to show induction of *PR* genes following 2,6-dichloroisonicotinic acid treatment and *P. syringae* infection. In addition, soybean plants showed activation of SAR following infection, suggesting the importance of *GmNPR1* for disease resistance in soybean.

An ortholog of *AtNPR1* has been cloned and characterized from peanut (*Arachis hypogeae*) that is mainly expressed in the roots and leaves^62^. While its overexpression in peanut is yet to be performed, co-overexpression of *BjNPR1* and a *defensin* homolog from *Trigunella foenum-graecum* (*Tfgd*) in peanut under control of the 35S promoter lead to resistance to *Aspergillus flavus*, a pathogen that can lead to production of dangerous mycotoxins such as aflatoxin. There was neither mycelial growth in the transgenic plants nor aflatoxin accumulation in their seeds. The transgenic plants also demonstrated varied levels of resistance to *Cercospora arachidicola* with reduced number of spots and delayed onset of disease^14^. This was a novel approach in overexpression of *AtNPR1* orthologs together with other resistance-related genes to obtain more resistance toward pathogens.

### *AtNPR1* orthologs in horticultural crops

Apple *AtNPR1* orthologs were cloned from *Malus pumila* (*MpNPR1*) or *Malus hupehensis* (*MhNPR1*) and overexpressed in apple (*Malus* × *domestica*)^5,63,64^. Constitutively high levels of *MpNPR1* enhanced resistance to three important diseases: fire blight (caused by *E*. *amylovora*), apple scab (caused by *Venturia inaequalis*), and cedar apple rust (caused by *Gymnosporangium juniperi-virginianae*)^5,63,64^. The transgenic plants did not exhibit detrimental morphological or developmental phenotypes, but they did constitutively upregulate *PR* genes. Increased transcript levels of *MhNPR1* in Fuji apple enhanced resistance to powdery mildew (caused by *P*. *leucotricha*)^64^. In transgenic tobacco *MhNPR1* induced expression of *PR* genes and contributed to salt and osmotic stress tolerance in addition to enhanced resistance to *B*. *cinerea*^65^. The tolerance to abiotic stress contrasted with what was observed in rice using the *AtNPR1* gene, which negatively affected tolerance to dehydration and salt stress^66^. Table 2 presents this example along with effects and outcomes of *AtNPR1* ortholog overexpression in various crops.

**Table 2:** Transgenic crop plants ectopically expressing *AtNPR1* orthologs

Plant species	*AtNPR1* ortholog	Defensive activity (caused by)	Abnormal phenotype	Reference
Apple	*MpNPR1*	Resistance to fire blight (*E*. *amylovora*) Resistance to apple scab (*V*. *inaequalis*) Resistance to cedar apple rust (*G*. *junipe*.*ri-virginianae*) Resistance to powdery mildew (*P*. *leucotricha*) Expression of *PR* genes	a	Malnoy et al.^5^ Malnoy et al.^63^ Chen et al.^64^
Apple	*MhNPR1*	Resistance to powdery mildew (*P*. *leucotricha*) Expression of *PR* genes	a	Chen et al.^64^
Canola	*BnNPR1*	Resistance to *P*. *syringae* Expression of *PR* genes	a	Potlakayala et al.^44^
Crabapple	*MhNPR1*	Tolerance to salt Tolerance to osmotic stress Resistance to fungus (*Botrytis cinerea*)	a	Zhang et al.^65,66^
Grapevine	*VvNPR1*.*1* *VvNPR1*.*2*	Resistance to downy mildew (*P*. *viticola*.) Resistance to powdery mildew (*E*. *necator*) Expression of PR proteins	Loss of apical dominance Death during acclimatization	Le Henanff et al.^2^^a^ Bergeault et al.^67^
Lily	*LhSorNPR1*	Enhanced resistance to *P*. *syringae* in tomato and Arabidopsis	a	Wang et al.^78^
Mustard	*BjNPR1*	Powdery mildew (*E*. *cruciferarum*).	a	Meur et al.^58^
Peanut	*BjNPR1*	Resistance to *Aspergillus flavus* Resistance to *Cercospora arachidicola*	a	Sundaresha et al.^14^
Potato	*StoNPR1*	Resistance to *Verticillium dahliae* Expression of *PR* genes	a	Deng-wei et al.^15^
Rice	*NH1*	Resistance to bacterial blight (*X*. *oryzae* pv. *oryzae)* Resistance to rice blast disease (*M*. *oryzae*) hypersensitive -like response after pathogen challenge Induction of benzothiadiazole, methyl jasmonate and ethylene defense molecules Expression of *PR* genes	Smaller plants Lower fresh weights and narrower or smaller leaves Tendency to precocious leaves senescence No development of additional tillers Spontaneously develop lesion-mimic spots Enhanced herbivore susceptibility Reduction of photosynthetic activities	Chern et al.^1^ Yuan et al.^8^ Sugano et al.^56^ Feng et al.^57^^a^
Tobacco	*NgNPR3*	Resistance to *A*. *alternata* Resistance to *P*. *solanacearum* Resistance to Potato Virus Y (PVY)	a	Zhang et al.^10^
Wheat	*ScNPR1*	Rye (*Secale cereale cv* Jingzhouheimai) version confers resistance to Fusarium Head Blight	a	Yu et al.^79^

^a^No abnormal/no reported phenotype

*AtNPR1* orthologs were also identified in grapevine (*Vitis vinifera*)^2^. The increased accumulation of *PR1* and *PR2* transcripts by *VvNPR1* both in *N*. *benthamiana* and *V*. *vinifera* strongly suggested that *VvNPR1* is also a component of the SA defense signaling pathway in grapevine. *VvNPR1*.*1* and *VvNPR1*.*2* are constitutively expressed, but their transcript levels may be increased with BTH treatment. These genes are functional when overexpressed in *N*. *benthamiana*, triggering the accumulation of *PR1* and *PR2* transcripts. To gain further information on *VvNPR1* activity in grape, the authors transiently expressed *VvNPR1*.*1* or *AtNPR1* in *V*. *vinifera* leaves. Interestingly, both *VvNPR1*.*1* and *AtNPR1* induced a stronger response to *P*. *viticola* inoculation. Later, Bergeault et al.^67^ provided further information about the *Vv*NPR1 proteins. *Vv*NPR1.1 and *Vv*NPR1.2 showed 52% and 37% identity to the AtNPR1 protein, respectively. All functional domains identified in AtNPR1 were conserved in the *Vv*NPR1 proteins.

Another functional characterization of *VvNPR1*.*1* and *VvNPR1*.*2*, including complementation of the Arabidopsis *npr1* mutant, confirmed *VvNPR1*.*1* as a functional ortholog of *AtNPR1*^68^. Transgenic grapevine plants overexpressing *VvNPR1*.*1* exhibited enhanced resistance to powdery mildew and induced accumulation of PR proteins. However, a loss of apical dominance related to *VvNPR1*.*1* overexpression was observed in all independent transformants. When ectopically expressed in the Arabidopsis *npr1-2* mutant, *VvNPR1*.*1* complemented the mutation, restoring normal plant growth, increasing SA concentration, and enhancing resistance to virulent *P. syringae* pv. *maculicola* infection.

With a similar approach, Zhang et al.^69^ demonstrated that overexpressing *VaNPR1*.*1*, an ortholog of *AtNPR1* in *V*. *aestivalis* cv. Norton, in Arabidopsis *npr1-1* mutant plants restored the accumulation of the *PR-1* transcript, although not completely. The results also showed that overexpression of *VaNPR1*.*1* in Arabidopsis plants increased tolerance to salinity, but not drought.

Complementation analyses also confirmed the functional conservation of *AtNPR1* orthologs in other plant species. Shi et al.^70^ isolated the *AtNPR1* ortholog from cacao (*Theobroma cacao*) and tested *TcNPR1* for complementation of the Arabidopsis *npr1-2* mutant. *TcNPR1* presented similar functions as *AtNPR1* and was able to partially complement the Arabidopsis *npr1-2* mutation. Like AtNPR1, the TcNPR1 protein was translocated to the nucleus after SA treatment and participated in the induction of *PR* gene expression, confirming its likely parallel role in defense response in cacao.

Similarly, two *AtNPR1*-like genes from banana (*MNPR1A* and *MNPR1B*) were isolated and overexpressed in Arabidopsis *npr1-2* mutant plants^71^. Overexpression of both genes restored the resistance of Arabidopsis *npr1-2* mutants to the biotrophic oomycete *Hyaloperonospora arabidopsidis*, the nectrotrophic fungus *B*. *cinerea*, and the hemi-biotrophic bacterial pathogen *P. syringae*. While the two genes possess different sequences, they are functionally indiscernible in complementation analyses.

Barsalobres-Cavallari et al.^72^ isolated the orthologous gene of *AtNPR1* from *Coffea arabica* (*CaNPR1*), representing a promising candidate for engineering resistance in coffee. Typical features of NPR1 proteins were found in *Ca*NPR1, including the BTB/POZ domain, an ankyrin repeat domain, and a nuclear localization signal. Surprisingly, transcript levels of *CaNPR1* were strongly activated by SA treatment but were not altered after pathogen infection.

The homologous genes, *GhNPR1* and *GhTGA2* from *Gladiolus hybridus*, one of the most economically important orchids, were isolated and functionally characterized^73^. Overexpression of *GhNPR1* in an Arabidopsis *npr1* mutant restored its basal resistance to *P*. *syringae* pv. *tomato* and silencing of *GhNPR1* resulted in enhanced susceptibility to *Curvularia* leaf spot caused by *Curvularia gladioli*.

Deng-wei et al.^15^ reported cloning of a *Solanum tovrum* (a wild eggplant) *NPR1* (*StoNPR1*) gene, which was responsive to salicylic acid and *Verticillium dahliae*. They overexpressed *StoNPR1* in *V. dahliae* sensitive potato plants and the transgenic lines demonstrated more resistance to this pathogen in comparison to wild-type and RNAi expressing lines. Furthermore, the expression of *isochorismate synthase1* (*ICS1*) and *PR1a* genes was discernably higher in *StoNPR1* overexpressing lines.

Other *AtNPR1* orthologs have been cloned and characterized from different crops. However, their functional analysis has yet been performed using gain or loss of function tests. For example, in avocado (*Persea* a*mericana*) three out of five *AtNPR1*-like genes showed a possible role in plant defense^74^. In papaya (*Carica papaya*), four homologous genes of *AtNPR1* were identified and their expression pattern in different tissues was evaluated^75^.

The tobacco (*Nicotiana glutinosa*) *NPR1* ortholog, *NgNPR3*, activates discrete signal transduction pathways. Zhang et al.^10^ showed that transgenic plants overexpressing this gene displayed enhanced resistance to *A*. *alternata, P*. *solanacearum*, and potato virus Y in a dose-dependent manner. The induction of *PR* genes after pathogen infection was higher in resistant plants and no obvious developmental defects were observed. Expression of the endogenous gene is induced by defense molecules (SA, INA, H_2_O_2_, and MeJA) and several pathogens (*P*. *solanacearum, P*. *parasitica, R*. *solani*, and *A*. *alternata*). The authors also reported that the NgNPR3 protein accumulates in the nucleus in response to SAR activators. The *Ng*NPR3 protein contains an ankyrin repeat domain and a BTB/POZ domain, which are motifs highly conserved among AtNPR1 and AtNPR1-like proteins. Taken together, these observations suggest a role for *NgNPR3* in induced defense responses in tobacco.

In addition to agronomic and horticultural applications, *AtNPR1* orthologs have been isolated and shown to confer effects in trees. Shao et al.^76^ isolated two *AtNPR1* homologous genes (*PtNPR1*.*1* and *PtNPR1*.*2*) from poplar (*Populus deltoids*). The *PtNPR1*.*1* and *PtNPR1*.*2* genes expressed in different tissues and responded to SA and MeJA with different time courses. While a *Pt*NPR1.1-GFP fusion protein was localized to the cytoplasm after being expressed in Arabidopsis mesophyll protoplasts, the authors concluded that these genes are promising candidates for engineering resistance to broad-spectrum pathogens in poplar. In sugarcane, an *AtNPR1* homolog (*ScNPR1*) was cloned and identified. *ScNPR1* was upregulated after SA or *Ustilago scitaminea* infection, but downregulated in response to MeJA or ET. Moreover, a higher accumulation of *ScNPR1* transcripts was found in the leaf and sheath tissues of resistant cultivars to *Ustilago scitaminea*^77^.

## Conclusion and prospects

High-value horticultural crop plants are constantly threatened by pests and pathogens. The emergence of epidemics, such as greening disease in citrus and laurel wilt in avocado, is a prominent reminder that new strategies are needed for disease tolerance. The loss of fumigants like methyl bromide and the projected phase-out of other fumigants leave fewer options for horticultural crop plant producers, meaning that genetic protection may be more feasible (and environmentally sustainable) than chemical protection.

There is significant interest in understanding the fundamental mechanisms of plant disease resistance, and the NPR1 protein has a well-defined role in this process. Whether through traditional breeding of *NPR1* alleles that affect disease presentation or defense gene expression, or transgenic installation of NPR1-modulating factors, this gene stands central in many designs for enhanced tolerance to biotic (and sometimes abiotic) challenges. There is potential for identification of specific alleles that could potentially be more active in various species, or perhaps pyramiding NPR1-related genes into a common genetic background. These approaches could speed molecular breeding efforts.

But all of these potential solutions must be approached conservatively. SAR induction is a complex system that involves multiple physiological and biochemical mechanisms and a substantial suite of genes. Constitutive activation of defense responses can exert deleterious effects on plant growth and development, and can impinge important processes relevant to crop production. Overexpression of the *AtNPR1* gene or its orthologs has been shown to enhance disease resistance in numerous plant species, including those with substantial commercial value. However, the same overexpressors also presented other negative attributes, such as yield loss, which could be an unacceptable trade-off to its implementation. Thus, understanding the physiological and molecular mechanisms of how resistance is elicited, and under which condition the resistance expression becomes deleterious for fitness, would greatly help crop disease resistance improvement.
